# KAP survey on toxoplasmosis among pregnant and post-partum women in Beni-Mellal region, Morocco

**DOI:** 10.11604/pamj.2025.52.15.38163

**Published:** 2025-09-12

**Authors:** Ilham Atif, Soukaina Kannane, Samia Boussaa, Oulaid Touloun

**Affiliations:** 1 Polyvalent Team of Research and Development, Polydisciplinary Faculty, Sultan Moulay Slimane University, Beni Mellal, Morocco,; 2 Higher Institute of Nursing and Health Techniques, Ministry of Health and Social Protection, Rabat, Morocco

**Keywords:** Toxoplasmosis, awareness, pregnant women, postpartum women, obstacles, Morocco

## Abstract

**Introduction:**

toxoplasmosis is an infectious disease caused by Toxoplasma gondii, a parasite capable of infecting all warm-blooded animals, including humans. If the infection is contracted during pregnancy, the parasite can be transmitted to the fetus, potentially leading to congenital anomalies. The present paper aimed to assess the level of awareness and knowledge of pregnant women regarding this disease, and to identify barriers to screening for toxoplasmosis during pregnancy in the province of Beni-Mellal, central Morocco.

**Methods:**

a cross-sectional study was conducted from 21st April to 14th July 2021, at the level of urban and rural health centers as well as the Regional Hospital in Beni-Mellal region, Morocco. A total of 412 questionnaires were completed by pregnant (64.6%) and post-partum women (35.4%).

**Results:**

only 27.2% of the women surveyed had received any information about toxoplasmosis. We found a significant association (P<0.05) between knowledge of toxoplasmosis and the woman's residence (urban or rural), profession, education level, and status (pregnant or postpartum). Regarding toxoplasmosis serology, 21.9% of the women were completely unaware of the existence of a toxoplasmosis test. Furthermore, 19.7% cited a lack of financial means as a barrier to testing, and 9.8% attributed the lack of information to healthcare providers' omission to clarify Toxoplasmosis and its consequences.

**Conclusion:**

this study highlights the pivotal role of effective communication in behavioral change. Therefore, it is crucial to implement an educational program targeting women of childbearing age about toxoplasmosis. Alongside this, ensuring free access to Toxoplasma serology testing is essential.

## Introduction

toxoplasmosis, caused by the coccidian parasite *Toxoplasma gondii*, is one of the most common diseases globally [1]. Humans primarily contract *T. gondii* by ingesting contaminated food or water, specifically from consuming raw or undercooked meat containing tissue cysts, or from oocysts present in contaminated environments due to infected cat faeces. Furthermore, vertical transmission from an infected mother to her fetus via the placenta is a critical route leading to congenital infection [2]. The degree of risk and severity of infection depends on the stage of pregnancy at which the mother contracts the infection; the earlier the infection occurs in pregnancy, the more severe the effects on the fetus. Indeed, early infection with *T. gondii* can result in spontaneous abortions [3] and stillbirths [4]. The newborns that survive may exhibit a range of severe consequences, including damage to the brain, liver [5], and spleen, alongside severe ocular infections that can progress to blindness [6]. Furthermore, the infection can affect auditory function [7] and lead to skull malformations such as hydrocephalus and microcephaly [8]. Some side effects of congenital *T. gondii* infection are not immediately apparent at birth, manifesting instead during childhood or adolescence. These delayed sequelae can include epilepsy [9], depression [10]. Autism spectrum disorder [11] and various long-term immune consequences [12].

Internationally, toxoplasmosis remains one of the most frequent parasitic infections; however, the seroprevalence is very variable depending on the lifestyle of the population and the geoclimatic conditions. The prevalence varies between 6.04% in Romani and 67.66% in Hungary. In North Africa, according to a review [13], the prevalence of this parasite in pregnant women is 28.6% in Algeria, 45.6% in Tunisia, 9.8% in Libya and 31.1% in Egypt. In Morocco, the seroprevalence varies between 28.88% in the Marrakech-Safi region and 43% in the Rabat region [14]. Introducing monthly prenatal screening and enhancing antenatal diagnosis have been shown to significantly reduce the rate of congenital *T. gondii* infection, leading to improved outcomes for infected children by three years of age [15]. The precise type and duration of toxoplasmosis treatment are contingent upon the severity of the infection and the potential for damage to vital organs. Typically, an approximately four-week course involving a combination of antibacterial and antiparasitic agents is employed, with pyrimethamine, sulfadiazine, and folinic acid serving as the primary therapeutic mainstays [16].

Preventive measures are necessary for the control of this infection in pregnant women, such as hygienic and dietary measures, sensitization and monthly serological surveillance in seronegative pregnant women [17]. The success of these measures depends on the level of awareness and knowledge of pregnant women. In this sense, a study carried out in the Beni Mellal-Khenifra Region [18] found that no pregnant women were tested for toxoplasmosis. A study conducted by Ouzennou *et al*. in Essaouira, Morocco in 2019 [19] revealed a significant gap in prenatal care: 96% of pregnant women had never undergone screening for anti-*Toxoplasma* antibodies. This finding is particularly concerning given that the test is systematic in prenatal check-ups throughout Morocco, which raises critical questions about why pregnant women aren't utilizing this available and crucial screening. Given the current paucity of data on this component within the province of Beni-Mellal, our study aims to assess the level of awareness and knowledge regarding toxoplasmosis among pregnant women in the region. Concurrently, we seek to identify the underlying reasons why women in this province do not access routine toxoplasmosis screening during pregnancy.

## Methods

**Figure 1:**
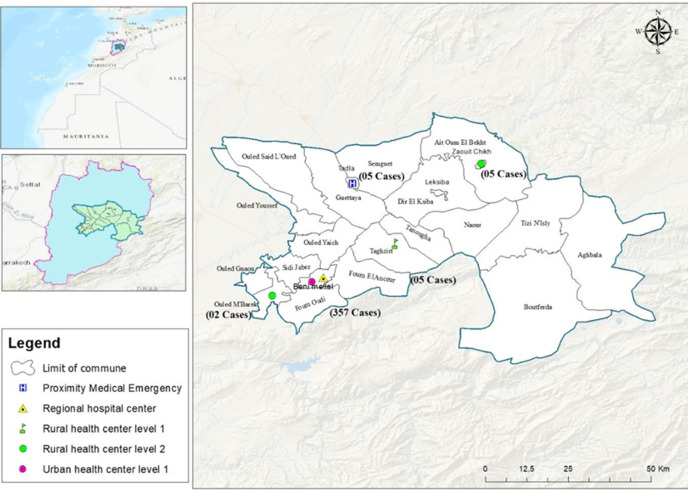
study area (Province of Beni Mellal)

**Study area:** this study took place in the Beni-Mellal province, centrally located in Morocco and spanning an area of 4528 km^2^ (Figure 1). In 2018, the province had an estimated population of 571,635 inhabitants. Healthcare infrastructure in the region includes two general hospitals, 13 urban health centers, 18 rural health centers, and 15 rural dispensaries [20]. In 2020, the number of deliveries in public institutions of the province and the number of post-natal consultations were 8,981 and 12,605, respectively [21].

**Study design and population:** a convenience sampling strategy was employed for participant recruitment. Between 21^st^ April and 14^th^ July 2021, a total of 412 participants were consecutively enrolled from a selection of health centers offering prenatal consultations and the regional hospital's maternity unit. These sites were chosen to capture a representative sample of pregnant and postpartum women seeking healthcare in the region. The minimum required sample size was calculated to be 373, based on the regional population of postpartum women (N=12605), with a 95% confidence interval and a 2% margin of error. To enhance statistical power and account for potential incomplete data or non-response, we aimed for a larger sample, ultimately enrolling 412 participants. Inclusion criteria for the study were pregnant and postpartum women who had not undergone previous serological testing for toxoplasmosis. The participants were approached by a research assistant at the study sites, informed about the research objectives, and invited to participate voluntarily.

**Data collection:** a structured questionnaire was constructed and used for the data collection. A pre-testing conducted on a random sample allowed the optimization of the instrument and to determine the time needed to complete the questionnaire too. The latest version of the questionnaire consisted of 22 closed-ended questions (each questionnaire took 7 to 10 minutes to be filed), divided into two sections, which aimed to collect from the respondents the following information: i) sociodemographic information: including social network; age; education; residence; occupational and employment status; origin; gestity, parity, medical coverage, pregnant or postpartum woman; term of the pregnancy; history of congenital malformation, pregnancy monitored, evolution of the pregnancy, notion of infection during pregnancy (13 questions); ii) knowledge of toxoplasmosis: have heard or receive information about toxoplasmosis and through which kind of information resources; knowledge about the parasite, enough explanations received; general clinical; diagnostic and prevention aspects of toxoplasmosis (8 questions); iii) reasons for not screening for toxoplasmosis.

**Statistical analysis:** the primary objectives of the statistical analysis were to: i) describe the sociodemographic characteristics and toxoplasmosis knowledge levels of the study population, and ii) identify associations between sociodemographic factors and the level of knowledge about toxoplasmosis. Data collected were primarily categorical (e.g., residence, education level, knowledge questions) and some were numerical (e.g. age). All data were entered and analyzed using IBM SPSS Statistics for Windows, Version 21.0 (Armonk, NY: IBM Corp.). Descriptive statistics were used to summarize the collected data. Categorical variables were presented as frequencies and percentages. Numerical data, such as age, were described using means and standard deviations or medians and interquartile ranges, as appropriate. To test for potential differences and associations, bivariate analyses were performed. The choice of statistical test was based on the nature of the variables being analyzed. Given the categorical nature of our data (e.g., associating residence with knowledge level), the Chi-square test (χ2) was used to assess the association between different sociodemographic characteristics and the participants' knowledge of toxoplasmosis. A significance level (p-value) of less than 0.05 (P < 0.05) was considered statistically significant. The results of the analysis will be presented using tables, which will include frequencies, percentages, and p-values for the statistical tests.

**Ethical approval:** data collection commenced following authorization from the Regional Directorate of Public Health and Social Protection (Ref. N°190/2021). Prior to questionnaire completion, participants were thoroughly apprised of the study's objectives, and their oral consent was duly acquired. The principles of anonymity, confidentiality, and privacy were rigorously upheld throughout the duration of the study.

## Results

**Sociodemographic characteristics of study population:** the study included 412 pregnant women aged 17 to 50, with a mean age of 28.3 ± 6.5 years. The majority were from urban areas (72.6%) and resided in Beni-Mellal (80.6%). Most participants were housewives (91.5%), 15.8% were illiterate, and just 48.1% had medical insurance. Additionally, 74.8% had fewer than three children. Of the 64.6% who were pregnant at the time of the study, 59.3% were in their third trimester. The remaining 35.4% of interviewees were in the postpartum period. Among them, 14 reported giving birth to infants with congenital malformations, including club foot, hand defects, and anal malformations. Most pregnancies (90.8%) were monitored, with 76% occurring in public health facilities. Additionally, 17.5% of pregnancies were classified as high-risk, and a quarter of pregnant women experienced anemia. Just under one-tenth (9.2%) of pregnant women reported an infection during pregnancy, predominantly genital (36.8%) or urinary (23.6%) infections, with one case of tuberculosis (Table 1).

**Table 1:** association between socio-demographic characteristics and awareness of toxoplasmosis among pregnant and postpartum women (N=412) surveyed in health facilities (April-July 2021)

Variable	Awareness of toxoplasmosis		N	X2	P-value
	Yes	No			
**Age range (Years)**			**410**	2.849	0.241
<25	36(8.8)	97(23.7)	133		
25-34	59(14.4)	141(34.4)	200		
≥ 35	15(3.6)	62(15.1)	77		;
**Residence**			**412**	3.266	0.047*
Urban	74(18)	225(54.6)	299		
Rural	38(9.2)	75(18.2)	113		
**Profession**			411	20.548	0.000*
Housewife	96(23.7)	291(70.5)	387		
Working	13(3.3)	7(1.5)	20		
Student	3(0.75)	1(0.25)	4		
**Educational level**			**412**	19.732	0.000*
Illiterate	6(1.5)	59(14.3)	65		
Elementary school	17(4.1)	40(9.7)	57		
Secondary school	30(7.2)	96(23.3)	126		
High school	39(9.5)	79(19.2)	118		
Higher education	20(4.9)	26(6.3)	46		
**Parity**			**412**	7.287	0.239
Nulliparous	2(0.5)	11(2.7)	12		
Pauciparous (1–2)	85(20.6)	217(52.6)	302		
Multiparous (≥ 3)	25(6.1)	72(17.5	97		
Status	&n		412	8.032	0.016*
Pregnant	82(20)	184(44.6)	266		
Post-partum	30(7.3)	116(28.1)	146		

* Statistically significant (p < 0.05)

**KAP survey:** overall, 27.2% of the women had prior awareness of toxoplasmosis, having either read, heard, or seen information about it. Among those aware, 49% received information from health professionals, 17% from family and friends, 12.5% from media, and 5% from internet research. Regarding specific knowledge, 40% of these women were unaware of when to undergo Toxoplasma serology testing. However, 55.4% reported knowing how the parasite is transmitted. Of these, 72.5% attributed infection to cat faeces contact, 11.3% to undercooked meat, and 6.5% to consuming contaminated vegetables and fruits. Furthermore, 66.1% of the women believed that the fetus could develop complications following toxoplasmosis infection, with 16.2% specifically mentioning hydrocephalus as a potential complication. A significant association (P < 0.05) was found between toxoplasmosis knowledge and residence (urban/rural), profession, educational level, and the woman's status (pregnant/postpartum). Specifically, 20.1% of pregnant women were aware of toxoplasmosis, compared to 7.4% of postpartum women, indicating significantly lower awareness in the latter group (P = 0.012) (Table 2).

**Table 2:** reasons for non-adherence to toxoplasmosis serology testing (N=412) among pregnant and postpartum women, surveyed between April and July 2021 in prenatal care facilities

Reasons	Number of women	%
Economic difficulties	177	19,7
Geographic inaccessibility	61	6,8
Lack of autonomy	67	7,5
Lack of awareness about the disease	197	21,9
Lack of awareness about the consequences	93	10,3
Belief that treatment is ineffective	35	3,9
Cultural barriers	126	14,0
unwillingness	35	3,9
Superstition	20	2,2
Health professional omission	88	9,8
**Total**	899	100

**Reasons for not screening for toxoplasmosis:** an analysis of the reasons women did not undergo toxoplasma serology revealed several key factors (Table 2). A significant proportion, 21.9%, were unaware of the existence of toxoplasmosis. Financial constraints affected 19.7% of women, while 14% cited their cultural background. Additionally, 10.3% were unaware of the disease's consequences, and 9.8% attributed the lack of screening to an omission by health professionals. Analysis revealed a significant correlation (P < 0.005) between educational level and both the woman's lack of autonomy in making screening decisions and omissions by health professionals (Table 3).

**Table 3:** association between educational level of pregnant and postpartum women and reasons for not undergoing toxoplasmosis screening (N=412)

Reasons		Illiterate	Elementary school	Secondary school	High school	Higher education	Total	P-value
**Economic difficulties**	Yes	35	51	53	27	11	177	0.067
	No	25	64	51	23	25	188	
**Geographic inaccessibility**	Yes	14	24	13	5	5	61	0.172
	No	46	91	90	45	30	302	
**Lack of autonomy**	Yes	11	31	19	9	0	70	0.001*
	No	49	84	84	41	35	293	
**Lack of awareness about the disease**	Yes	36	66	61	21	13	197	0.051
	No	24	49	42	29	22	166	
**Lack of awareness about the consequences**	Yes	15	37	25	10	6	93	0.299
	No	45	78	78	40	29	270	
**Belief that treatment is ineffective**	Yes	3	9	11	10	2	35	0.094
	No	57	106	92	40	33	328	
**Cultural barriers**	Yes	24	40	36	25	1	126	0.000*
	No	36	75	67	25	34	237	
**Unwillingness**	Yes	4	12	12	5	3	36	0.873
	No	56	103	91	45	32	327	
**Superstition**	Yes	2	10	6	2	0	20	0.148
	No	58	105	97	48	35	343	
**Health professional omission**	Yes	18	32	19	5	14	88	0.005*
	No	42	82	84	45	21	274	

* Statistically significant (p < 0.05) &n

## Discussion

Toxoplasmosis is a public health problem that affects both humans and animals; unfortunately, pregnant women are a group at risk for this parasite [22]. A wide variety of health problems result from this infection: spontaneous abortion, prematurity, and perinatal death. In addition, sequelae on the developing fetus's brain and eyes, known as the characteristic triad of congenital toxoplasmosis (chorioretinitis, hydrocephalus, and cerebral calcifications) [4] and even long-term sequelae are possible for affected newborns. In Morocco, *T. gondii* seroprevalence in pregnant women is considered a serious public health problem. This prevalence can reach a very high rate of 51% [23]. To conduct this research, a population of 412 women participated in this study, of which 64.6% of the women were pregnant and 35.4% were in the post-partum period. 48.5% of the participants are between 25and 34 years old, representing a predominantly young adult age group. This result is observed by comparing it with a study done in Casablanca, Morocco [24], where the most dominant age group of mothers was 30-35 years old. Moreover, the results of a study found that the mother´s age significantly influenced the seroprevalence rate [25].

Concerning the place of residence, the majority of mothers are of rural origin, which raises the question of geographical accessibility of the rural population for care. Correspondingly, another study carried out in Rabat, Morocco, found the prevalence of *T. gondii* to be 55% in rural areas compared to 38.6% in urban areas [26]. Throughout this study, there was a significant association between the knowledge about *T. gondii* and the residence area (urban or rural). For the level of education, it was found that 46.4% of these pregnant women are illiterate or have just an elementary-level school, which influences the level of knowledge about the disease. The illiteracy rate in the province of Beni-Mellal is 45.2% among women [20]. Moreover, the low educational level is mentioned as a risk factor of toxoplasmosis in Morocco [23]. Morocco's demographic transition, marked by a declining total fertility rate (2.2 nationally, 2.1 in our study region) [20], led to 74.8% of our respondents having fewer than three children. This is significant because parity has been shown to influence *T. gondii* seroprevalence in Morocco [26].

Reflecting international and national recommendations, 90.8% of pregnancies in our study were monitored. A significant majority (76%) utilized public health structures, largely due to their accessibility and free services. In Morocco, monthly screening for *T. gondii* is recommended for seronegative pregnant women. The Moroccan Ministry of Health and Social Protection recommends the use of enzyme-linked immunosorbent assay (ELISA) for serological testing. However, this crucial test is unavailable in public hospitals and birthing centers within the study area. Consequently, all women were forced to seek testing at private laboratories. This situation places an additional economic burden on the population, which is particularly challenging given the high poverty rate in the region [20]. In addition, the low adherence of these families to health coverage further compounds this issue; in our study, only 48.1% of pregnant women had medical insurance. More than half of the mothers were in the third trimester and unfortunately missed several screening opportunities. This finding is in line with a study [19] which confirmed that 96% of pregnant women have never done any screening of *T. gondii* antibodies. Our study revealed a very low level of toxoplasmosis knowledge, with only 27.2% of women having encountered information on the disease. This is lower than a Casablanca, Morocco study (41.2%) [15,25], but higher than one in Essaouira, Morocco, where only 22 out of 600 women showed good knowledge [19].

Knowledge of the mode of transmission of this infection is the key to prevention of *T. gondii* infection. In the present study, 55.4% of the women reported knowing how to get the parasite. Of these, 72.5% attributed the infection to contact with cats. Similarly, a study conducted in Iran [27] showed that 31.3% of pregnant women were aware of close contact with cats. Furthermore, in our study, only 11.3% of the respondents thought that undercooked meat causes this disease, and 6.5% of them recognized the risk from consuming contaminated fruits and vegetables. This is significantly different from an Iranian study [27], where 47.5% of pregnant women associated disinfecting produce with preventing intestinal parasitic infections. Our study found that no women were aware of *T. gondii* infection through soil contact. This lack of awareness contrasts with a study in the Netherlands, which identifies soil as a significant source of *T. gondii* exposure [28]. Similarly, an Essaouira, Morocco study (2019) ranked direct soil contact as the primary risk factor for Toxoplasma transmission, ahead of poor hygiene and raw meat handling [19]. Furthermore, a systemic review in Morocco [23] highlighted direct soil contact, low awareness, and low education levels among pregnant women as major contributors to high *T. gondii* prevalence.

Pregnant women in our study were aware of the fetus' risk from toxoplasmosis (66.1%), yet only 16.2% knew hydrocephalus was a complication, indicating a broader knowledge gap on congenital malformations. This is significant, as 14 participants reported births with congenital malformations. While studies link prenatal *T. gondii* to severe issues like hydrocephalus, microcephalous, and various other defects [4], one study found no connection to hearing or vision problems [7]. Crucially, half of the participants received information from health professionals, whose advice is known to reduce maternal anxiety and vertical transmission risk [29]. In our study, 19.7% of women did not have the financial means to benefit from the serological test. In Germany, a research team found that participation in toxoplasmosis screening was positively associated with the socioeconomic status of pregnant women, particularly when they had a high time burden and family responsibilities [30]. The results of this study cannot be generalized, as each region of Morocco has its own demographic and economic characteristics.

**Limitations:** the results of this study cannot be generalized to all of Morocco. Each region has its own unique demographic and economic characteristics that can significantly influence non-adherence to serology testing. For instance, geographic inaccessibility is likely a more prominent risk factor in rural or mountainous regions than in the urban context where our study was conducted. Furthermore, cultural barriers and awareness levels about the disease may vary according to regional specificities. Future multicenter studies including participants from different regions would be necessary to better assess the impact of these factors on toxoplasmosis prevention at a national level.

## Conclusion

Toxoplasmosis infection during pregnancy poses a significant public health threat to reproductive health. This study was the first in Morocco's Beni-Mellal region to assess pregnant women's knowledge of toxoplasmosis and the barriers to screening. Our findings reveal a widespread lack of knowledge regarding this infection. Participants primarily associated transmission with cat contact, with few aware that undercooked meat or contaminated fruits and vegetables could also cause the disease. The study identifies ignorance about the disease's existence and insufficient financial resources as the primary barriers to toxoplasmosis serology screening among women. This research underscores the critical role of communication in driving behavioral change. We propose several recommendations: systematically organize education and awareness sessions for girls and women of childbearing age, both before and during conception, delivered by qualified health personnel; revitalize existing parent classes established by the Moroccan Ministry of Health and Social Protection; provide training and supervision for health professionals; ensure free toxoplasma serology; and engage mass media in public awareness campaigns for congenital toxoplasmosis.

### What is known about this topic

- Congenital toxoplasmosis can lead to malformations in the fetus;
- Toxoplasmosis serology is a recommended prenatal test in Morocco but not free of charge.

### What this study adds

- This study reveals that only 27.2% of surveyed women had received any information about toxoplasmosis;
- A significant portion (21.9%) were completely unaware of the existence of a toxoplasmosis test;
- Financial barriers and lack of healthcare provider clarification were identified as key deterrents to testing and information.
